# AMG2850, a potent and selective TRPM8 antagonist, is not effective in rat models of inflammatory mechanical hypersensitivity and neuropathic tactile allodynia

**DOI:** 10.1007/s00210-015-1090-9

**Published:** 2015-02-10

**Authors:** Sonya G. Lehto, Andy D. Weyer, Maosheng Zhang, Beth D. Youngblood, Judy Wang, Weiya Wang, Patrick C. Kerstein, Carl Davis, Kenneth D. Wild, Cheryl L. Stucky, Narender R. Gavva

**Affiliations:** 1 Department of Neuroscience, Amgen Inc, One Amgen Center Dr, Thousand Oaks, CA 91320-1799 USA; 2 Department of Pharmacokinetics and Drug Metabolism, Amgen Inc, One Amgen Center Dr, Thousand Oaks, CA 91320-1799 USA; 3 Department of Cell Biology, Neurobiology and Anatomy Medical College of Wisconsin, 8701 Watertown Plank Road, Milwaukee, WI 53226 USA

**Keywords:** TRPM8, AMG2850, Pain, Inflammatory, Neuropathic, Hypersensitivity, Small molecule, Therapeutic, Rat

## Abstract

TRPM8 has been implicated in pain and migraine based on dorsal root- and trigeminal ganglion-enriched expression, upregulation in preclinical models of pain, knockout mouse studies, and human genetics. Here, we evaluated the therapeutic potential in pain of AMG2850 ((*R*)-8-(4-(trifluoromethyl)phenyl)-*N*-((*S*)-1,1,1-trifluoropropan-2-yl)-5,6-dihydro-1,7-naphthyridine-7(8H)-carboxamide), a small molecule antagonist of TRPM8 by in vitro and in vivo characterization. AMG2850 is potent in vitro at rat TRPM8 (IC_90_ against icilin activation of 204 ± 28 nM), highly selective (>100-fold IC_90_ over TRPV1 and TRPA1 channels), and orally bioavailable (*F*_po_ > 40 %). When tested in a skin-nerve preparation, AMG2850 blocked menthol-induced action potentials but not mechanical activation in C fibers. AMG2850 exhibited significant target coverage in vivo in a TRPM8-mediated icilin-induced wet-dog shake (WDS) model in rats (at 10 mg/kg p.o.). However, AMG2850 did not produce a significant therapeutic effect in rat models of inflammatory mechanical hypersensitivity or neuropathic tactile allodynia at doses up to 100 mg/kg. The lack of efficacy suggests that either TRPM8 does not play a role in mediating pain in these models or that a higher level of target coverage is required. The potential of TRPM8 antagonists as migraine therapeutics is yet to be determined.

## Introduction

The transient receptor potential melastatin 8 (TRPM8) is a nonselective cation channel known as the “cool receptor” stemming from early work describing the cooling-activated current (Reid and Flonta 2001). Later, the localization of TRPM8 in subpopulations of small-diameter, cold-sensitive peripheral sensory neurons confirmed its activation by cool temperatures or compounds known to evoke cooling sensations such as menthol and icilin (McKemy et al. 2002; Peier et al. 2002; Nieto-Posadas et al. 2011). Further evidence of the role of TRPM8 in thermal sensation and even thermoregulation was generated with TRPM8 knockout (KO) mice. TRPM8 KO mice do not show the normal warm temperature preference that is typical in mice (Dhaka et al. 2006; Bautista et al. 2007; Colburn et al. 2007; Dhaka et al. 2007; Knowlton et al. 2010; Knowlton and McKemy 2011) nor do they show normal adaptive responses to cold or menthol-induced thermoregulatory challenges (Tajino et al. 2007, 2011; Almeida et al. 2012; Gavva et al. 2012).

Recent genome-wide association studies (GWAS) involving migraine patients have demonstrated a strong association of TRPM8 with migraine. Single nucleotide polymorphs (SNPs) within the TRPM8 gene have consistently been found to show a protective association with migraine in multiple studies as well as in a meta-analysis of all migraine GWAS suggesting that TRPM8 modulators may act as migraine therapeutics (Chasman et al. 2011; Schurks 2011; Freilinger et al. 2012; Anttila et al. 2013; Esserlind et al. 2013; Chasman et al. 2014).

Besides interest in TRPM8 as a temperature sensor and target for migraine, it has also been considered as a potential target for chronic pain based on results in KO mice, receptor expression, and early pharmacology (Daniels and McKemy 2007; Stucky et al. 2009; Liu and Qin 2011; Malkia et al. 2011; McCoy et al. 2011; Almaraz et al. 2014). KO mice have also been used to demonstrate a significant role for TRPM8 in the inflammatory and neuropathic injury-related increases in cold sensitivity (Colburn et al. 2007; Xing et al. 2007; Descoeur et al. 2011) and also that TRPM8 is required for cooling-evoked analgesia (Proudfoot et al. 2006; Dhaka et al. 2008). TRPM8 expression in free nerve terminals of skin, and localization in Aδ and C neurons in the dorsal root and trigeminal ganglia, is consistent with a role in both thermosensation as well as cold hypersensitivity (McKemy et al. 2002; Park et al. 2006; Proudfoot et al. 2006; Fleetwood-Walker et al. 2007; Takashima et al. 2007; Belmonte et al. 2009; Knowlton and McKemy 2011; McCoy et al. 2011).

Although delivery of a therapeutic that addresses the clinical need for reduction of cold hypersensitivity in conditions such as joint pathology or chemotherapy-induced peripheral neuropathy would be beneficial for a large number of patients, a drug that additionally mitigates the mechanical hypersensitivity and spontaneous pain in these disorders would have even broader clinical utility. Thus, TRPM8 upregulation in preclinical models of pain that are characterized by mechanical hypersensitivity and ongoing pain, including chronic constriction injury, complete Freund’s adjuvant (CFA) model, and oxaliplatin-induced hypersensitivity (Gauchan et al. 2009), implicates TRPM8 as an ideal target for mediating not only cold-evoked hypersensitivities but also inflammatory- or neuropathic-induced pathophysiology itself. As interest increases in TRPM8 as a more widespread pain target (Malkia et al. 2009; Ferrer-Montiel et al. 2012; Tamayo et al. 2012; Almaraz et al. 2014; Horne et al. 2014), a large number of patents for potent TRPM8 antagonists are emerging.

To assess the therapeutic potential of TRPM8 antagonists for chronic pain, we characterized AMG2850 as a potent, selective, orally bioavailable antagonist of TRPM8 and profiled it in models of inflammatory and neuropathic pain.

## Methods

### Compounds and reagents

AMG2850, which was synthesized at Amgen Inc (Thousand Oaks, CA), resulted from an internal medicinal chemistry effort (Horne et al. 2014). All the cell culture reagents were purchased from Invitrogen (Carlsbad, CA).

### In vitro characterization

#### Luminescence readout assay for measuring intracellular calcium

Stable Chinese hamster ovary (CHO) cell lines expressing rat TRPA1, rat TRPM8, rat TRPV3, and human TRPV4 were generated using the tetracycline-inducible T-REx™ expression system from Invitrogen (Carlsbad, CA) and a stable CHO cell line expressing rat TRPV1 was generated using a constitutive expression system (Klionsky et al. 2007). To enable a luminescence readout based on intracellular increase in calcium (Le Poul et al. 2002), each cell line was also co-transfected with pcDNA3.1 plasmid containing jelly fish aequorin cDNA. Twenty-four hours before the assay, cells were seeded in 96-well plates and all TRP channel expression, except for TRPV1, was induced with 0.5 ug/mL tetracycline. On the day of the assay, culture media were removed and cells were incubated for 2 h with pH 7.2 assay buffer (F12 containing 30 mM 4-(2-hydroxyethyl)-1-piperazineethanesulfonic acid (HEPES) for TRPV1, TRPA1, TRPM8, and TRPV3; F12 containing 30 mM HEPES, 1 mM CaCl_2_, and 0.3 % BSA for TRPV4) containing 15 uM coelenterazine (P.J.K GmbH, Germany). AMG2850 was added 2.5 min prior to the addition of an agonist. Luminescence was measured by a CCD camera-based FLASH-luminometer built by Amgen, Inc. The following agonists were used to activate TRP channels: 0.5 uM capsaicin for TRPV1, 80 uM allyl isothiocyanate (AITC) for TRPA1, 1 uM icilin or cold buffer (12 °C) for TRPM8, 200 uM aminoethoxydiphenyl borate (2-APB) for TRPV3, and 1 uM 4α-phorbol 12,13-didecanoate (4α-PDD) for TRPV4. Antagonist IC_50_ values were calculated using GraphPad Prism 4.01 (GraphPad Software Inc, San Diego, CA).

#### Agonist-induced ^45^Ca^2+^ uptake assay

Two days prior to the assay, cells were seeded in Cytostar 96-well plates (Amersham) at a density of 20,000 cells/well. The activation of TRPM8 was followed as a function of cellular uptake of radioactive calcium (^45^Ca^2+^) upon cold or icilin stimulation. To determine the ability to block agonist activation of TRPM8, AMG2850 was incubated with CHO cells expressing the TRP channel for 2 min before the addition of agonist and ^45^Ca^2+^ and cells were washed after a further incubation of 2 min to determine the ^45^Ca^2+^ uptake. All antagonist ^45^Ca^2+^ uptake assays were conducted as reported previously (Gavva et al. 2007), with a final ^45^Ca^2+^ concentration of 10 μCi/mL. Radioactivity was measured using a MicroBeta Jet (PerkinElmer, Inc.). Data were analyzed using GraphPad Prism 4.01 (GraphPad Software Inc, San Diego, CA).

### Pharmacokinetics in plasma and brain

Plasma pharmacokinetics of each compound in male Sprague-Dawley rats (*n* = 3 animals per study) were determined after intravenous dosing at 2 mg/kg in 100 % dimethylsulfoxide (DMSO) or after administration of 5 mg/kg by oral gavage with test article formulated in 5 % Tween 80 in OraPlus®. At designated time points, blood was collected via the femoral artery and processed for plasma by centrifugation. Plasma was then transferred into a 96-well container and stored in a freezer maintained at −70 °C. Similar bioanalytical methods were used to determine plasma and brain exposure in animals tested in pharmacodynamic and pain models. Brain samples were homogenized with a 4:1 ratio of water (mL) for every gram of brain tissue. All analytical methods utilized protein precipitation, with the addition of a structurally similar compound to function as an internal standard. Calibration standards were prepared from a 1 mg/mL stock solution of each compound in DMSO with a serial dilution into a blank matrix (plasma or brain homogenate). Liquid chromatography with tandem mass spectrometry was used for the quantitation of each compound in rat plasma and brain. Non-compartmental pharmacokinetic analysis of plasma concentrations was conducted using WinNonlin Enterprise v.5.1.1 (Pharsight Corporation, Mountain View, CA). Brain uptake was calculated as a ratio of concentration in whole brain tissue to that in plasma taken from the same animal at approximately the same time.

### Plasma protein binding

The unbound fraction in plasma was determined by ultracentrifugation. Test compound (5 ug/mL) was incubated in plasma from male rats at 37 °C for 15 min. Samples were transferred to a centrifuge tube and centrifuged at 16,128×*g* for 3 h at 37 °C. After protein precipitation using acetonitrile containing an internal standard (2:1 acetonitrile to plasma), supernatants were dried under a stream of nitrogen gas, with residues reconstituted in methanol/water (50:50, *v*/*v*) prior to analysis by liquid chromatography with tandem mass spectrometry. The concentration was determined from a linear regression of peak area ratios (analyte peak area/internal standard peak area) relative to calibration standards. The unbound fraction in plasma (*C*_u_) was calculated as a ratio of the concentration measured relative to the nominal concentration.

### Skin nerve preparation and fiber analysis

Adult C57BL/6 mice were obtained from Jackson Laboratories and used for ex vivo skin-nerve preparations. Mice were allowed to acclimate to the animal facility at the Medical College of Wisconsin, an Association for the Assessment and Accreditation of Laboratory Animal Committee (AALAC)-accredited facility, for approximately 1 week following shipment. Animals were housed with up to four other cagemates and had ad libitum access to food pellets and hyper-chlorinated water. Mice were housed on a 14:10 h light-dark cycle in cages containing aspen bedding and paper nesting material. Room temperatures averaged 21 °C. Prior to use, animals were briefly anesthetized with inhaled isoflurane and then sacrificed via cervical dislocation. All procedures were approved by the Institutional Animal Care and Use Committee at the Medical College of Wisconsin.

The mouse saphenous skin-nerve in vitro preparation (Koltzenburg et al. 1997; Stucky et al. 1999) was used for electrophysiological recordings of cutaneous terminals of primary afferent fibers in situ. Recordings were conducted on adult male C57BL/6 mice, aged 7–18 weeks. Mice were anesthetized with isoflurane and sacrificed by cervical dislocation. The saphenous nerve and skin from the medial dorsum of the hind paw were then rapidly dissected free and placed corium side up into a bath superfused with oxygen-saturated synthetic interstitial fluid containing (in mM) 123 NaCl, 3.5 KCl, 0.7 MgSO_4_, 1.7 NaH_2_PO_4_, 2.0 CaCl_2_, 9.5 sodium gluconate, 5.5 glucose, 7.5 sucrose, and 10 HEPES, 290 mOsm, at pH 7.45 ± 0.05, and temperature 32.0 ± 0.5 °C. The saphenous nerve was desheathed and teased into fine filaments for extracellular recordings, as described (Kwan et al. 2009). Single afferent units were identified using a mechanical search stimulus (blunt glass rod). C fibers were identified by conduction velocities slower than 1.2 m/s. Once a unit with a signal to noise ratio >2 was identified, the mechanical threshold was determined using calibrated von Frey filaments (range, 0.44–147.0 mN).

For treatment with TRPM8 inhibitors, the receptive field of each C fiber was isolated with a metal ring (4-mm diameter) sealed to the skin and incubated in vehicle (0.1 % DMSO or 0.003 % DMSO) or 10 μM AMG2850 for 10 min. AMG2850 was dissolved to the final concentration in 0.1 % DMSO. A baseline recording was taken during the final 2 min of compound incubation to measure any ongoing action potentials.

For mechanical stimulation in the presence of test compounds, the receptive field of each C fiber was tested with a series of increasing mechanical forces (5, 10, 20, 40, 100, 150, 200, 245 mN; 10-s sustained force; 1-min inter-stimulus interval). For chemical stimulation, the receptive field of each C fiber was stimulated with 300 μM menthol in the presence of the respective compound and action potentials were recorded for 2 min. C fibers were considered to be responsive to menthol if they fired at least three action potentials above baseline during the 2-min incubation period.

Wave forms of action potentials evoked by mechanical stimuli were saved on an oscilloscope for comparison of shape and profile. Data were collected using a Powerlab 4.0 system and LabChart software (ADInstruments, Colorado Springs, CO) and saved for off-line analysis. Action potentials were discriminated and counted off-line using a spike histogram software extension. For C fibers that exhibited ongoing activity in the presence of TRPM8 inhibitors, the frequency of action potentials during baseline was subtracted from action potentials evoked during mechanical or menthol stimulation. The operator was blinded to chemical treatment applied to each receptive field. All data was also analyzed in a blinded manner.

### In vivo pharmacodynamic and pain models

Adult male Sprague-Dawley rats weighing 220–300 g (Harlan, San Diego) were cared for in accordance to the *Guide for the Care and Use of Laboratory Animals*, *8th Edition* (National Research Council Committee for the Update of the Guide for the C and Use of Laboratory A 2011). Animals were group housed at an Association for Assessment and Accreditation of Laboratory Animal Committee-accredited facility in non-sterile ventilated micro-isolator housing on corn cob bedding. All research protocols were approved by the Institutional Animal Care and Use Committee. Animals had ad libitum access to pelleted feed (Harlan Teklad 2020X, Indianapolis, IN) and water (on-site generated reverse osmosis) via automatic watering system. Animals were maintained on a 12:12 h light-dark cycle in rooms at 21 ± 3 °C, 50 ± 20 % room humidity, and had access to enrichment opportunities (nesting materials and plastic domes). All animals were sourced from approved vendors who meet or exceed animal health specifications for the exclusion of specific pathogens (i.e., mouse parvovirus, Helicobacter). Animals were allowed at least 1-week acclimation to the facility prior to any procedures. Following completion of behavioral or blood pressure measurements, animals were euthanized with carbon dioxide followed by immediate blood collection via cardiac puncture for pharmacokinetic analysis. All behavioral data was scored by an observer blind to dosing condition or through an automated device.

#### Icilin-induced wet-dog shake (WDS) model

The TRPM8 antagonist (1, 3, or 10 mg/kg) or vehicle control (5 % Tween 80 in OraPlus®) was administered p.o. 90 min prior to administration of icilin (0.5 mg/kg, 1 ml/kg, i.p., 100 % PEG 400). The number of shakes exhibited by the rats (wet-dog shakes; WDS) was counted for a duration of 30 min post icilin administration (Werkheiser et al. 2006). Rats were viewed through transparent Plexiglas® observation cylinders that were placed on a custom, opaque, plastic apparatus such that one rat could not view another rat. The 0.5 mg/kg dose of icilin was chosen based on an in-house dose-response effect over 0.3–1.0 mg/kg in which nonlinear dose-response curve analysis yielded a goodness of fit *r* ^2^ of 0.84, and the resulting ED_90_ was 0.46 mg/kg (0.28–0.75; data not shown).

#### Cold-induced increase in blood pressure—cold pressor test (CPT)

Rats were treated with the TRPM8 antagonist (0.3, 1, or 3 mg/kg) or vehicle control (5 % Tween 80 in OraPlus®) p.o. 2 h prior to anesthesia with sodium pentobarbital at 60 mg/kg i.p. Blood pressure was recorded for 5 min for “pre-cold baseline” and an additional 5 min during immersion of the paws and ventral half of the body in ice water. Mean blood pressure (MBP) was derived electronically using Digi-Med Blood Pressure Analyzer (Model 400). Plasma was collected through the artery catheter immediately after the cold pressor test for pharmacokinetic analysis. Percent increase from baseline and percent inhibition attributed to treatment with test compound were then determined using the following formula: (1) percent increase from baseline (mmHg) cold pressor effect = [(cold-evoked change in MBP)/MBP pre-cold] × 100; (2) percent inhibition of cold pressor effect = [1 − (cold-evoked change in MBP post-test compound/cold-evoked change in MBP post-vehicle)] × 100.

#### Open field activity

Rats were habituated in a reversed light cycle room in home cages for at least 1 week and acclimated to the testing room for 1 h prior to dosing. TRPM8 antagonist (10, 30, or 100 mg/kg) or vehicle (5 % Tween 80 in OraPlus®) was injected 2 h before placing an animal in the open-field apparatus. Open-field activity was measured using a system that counts interruptions of a set of photobeams for the course of 60 min (Kinder Scientific, Poway, CA). To begin a session, animals were removed from the home cage and placed individually into an independent Plexiglas box (41 cm L × 41 cm W × 38 cm H) surrounded by a frame consisting of 32 photocells (16Y and 16X) that track the movement of the animal. Photobeam breaks were used as an indication of activity and were measured as the following parameters per minute: basic movements (beam breaks), distance traveled (cm), time spent (s), and number of repetitive beam breaks (i.e., stereotypic movement). Chlordiazepoxide, which consistently elicits a decrease in open field activity, was used as a positive control. Chlordiazepoxide was formulated in saline at 5.6 mg/kg i.p. and dosed 30 min before the start of the open field assay.

#### CFA-induced hypersensitivity

Rats were habituated in a reversed light cycle room for at least 1 week and to the testing room for 1 h prior to dosing. To begin the study, rats received an injection of CFA into the left hind paw (100 μL of 0.1 % CFA in saline) based on previous demonstration that this causes a decrease in rearing. Since this rearing deficit can be significantly reversed by treatment with indomethacin, it is presumed to represent mechanical and/or tactile hypersensitivity and rats are rearing less in order to avoid weight bearing on the inflamed hindpaw (Youngblood et al. 2008). Twenty-one hours after the CFA injection, animals were treated (p.o.) with vehicle (5 % Tween 80 in OraPlus®) or TRPM8 antagonist. Two hours after drug dosing (23 h after CFA injection), rats were placed in the same Kinder Scientific open field boxes as described above but with customized prickly floor inserts placed inside. Rearing time and rearing counts were measured for 60 min. Indomethacin at a dose of 1 mg/kg dosed p.o. in 5 % Tween 80 in OraPlus® was chosen as a positive control based on reliable efficacy demonstrated in previous experiments (Youngblood et al. 2008).

#### Sciatic nerve ligation (SNL)-induced tactile allodynia

SNL surgery was performed using aseptic surgical techniques and a stereomicroscope (Kim and Chung 1992). Spinal nerve injury was caused by ligating the left L5 and L6 spinal nerves, with special care to avoid damage to the L4 spinal nerve or surrounding area. More specifically, under gaseous anesthesia with a mixture of O_2_ and isoflurane (3 % for induction and 2 % for maintenance), skin was excised and the longissimus lumborum muscle, part of articular processes (L4-S1), and the fascia above L6 spinal nerve were carefully removed. This procedure provided a clean and spacious working area to enable complete resection of the L6 transverse process and to separate the L5 spinal nerve from the L4 spinal nerve without damage to L4. The L5 and L6 spinal nerves were each tightly ligated with 6-0 silk thread and then L5 was cut. The entire surgery procedure beginning from anesthesia and ending with wound clipping of the outside skin lasted 15 min or less.

### Surgical procedure

*Behavioral testing* Two weeks post surgery, mechanical sensitivity was measured by determining the median 50 % foot withdrawal threshold for von Frey filaments using the up-down method (Chaplan et al. 1994). Rats were placed under a plastic cover (9 × 9 × 20 cm) on a metal mesh floor. von Frey filaments (Semmes-Weinstein monofilaments from Stoelting) were applied to the middle glabrous area between the footpads of the plantar surface of the injured hind paw. This plantar area was touched with a series of nine recently calibrated von Frey filaments with approximately exponentially incremental bending forces (von Frey filament numbers 3.61, 3.8, 4.0, 4.2, 4.41, 4.6, 4.8, 5.0, and 5.2; equivalent to 0.41, 0.63, 1.0, 1.58, 2.51, 4.07, 6.31, 10, and 15.8 g). The von Frey filament was presented perpendicular to the plantar surface with sufficient force to cause slight bending and held for approximately 3–4 s. To avoid possible reflex responses, only abrupt withdrawal of the foot accompanied by pain indicative behaviors (namely paw flinching, shaking, or licking for more than 2 s) was recorded as a response. Any post-surgery rat that displayed a mechanical threshold of more than 3.16 g or less than 0.7 g was eliminated from the study. After measuring basal threshold, animals were treated (p.o.) with vehicle (5 % Tween 80 in OraPlus®) or TRPM8 antagonist (AMG2850 at 100 mg/kg), or gabapentin (200 mg/kg; Sigma, St. Louis). The measurement of the tactile threshold was reassessed at 1 and 2 h after drug administration in the same animals.

#### Statistical analyses

In vivo IC_50_ and IC_90_ estimates using WDS model data were determined by fitting a sigmoidal dose-response curve to individual animal number of WDS versus resulting plasma concentration. Behavioral and electrophysiological data are expressed as mean ± standard error of the mean (S.E.M.). Results were analyzed using one-way analysis of variance (ANOVA) with Dunnett’s multiple-comparisons post-hoc test for significance relative to vehicle. Since the von Frey filament set was calibrated on a logarithmic scale by the vendor (Stoelting), our selection of nine filaments for the up-down method was also based on near-equal logarithmic intervals (Dixon 1980), and because it is our experience that variability noticeably increases with threshold value and also variances are statistically different using Bartlett’s test of equal variances, thus violating the assumptions of ANOVA, data were analyzed following logarithmic transformation prior to statistical analysis. Actual gram values are plotted on a logarithmic scale *Y*-axis of the figures for convenience. Statistical calculations and graphs were made using GraphPad Prism 5.01 (GraphPad Software Inc, San Diego, CA). Percent responders to menthol stimulation in the ex vivo skin-nerve preparation were compared under vehicle and antagonist conditions through the use of Fisher’s exact test. Responses of C fibers to increasing mechanical forces under vehicle or antagonist conditions were compared using a two-way ANOVA with a Bonferonni post-hoc analysis.

## Results

### AMG2850 acts as a potent and selective TRPM8 antagonists in vitro

**Fig. 1:**
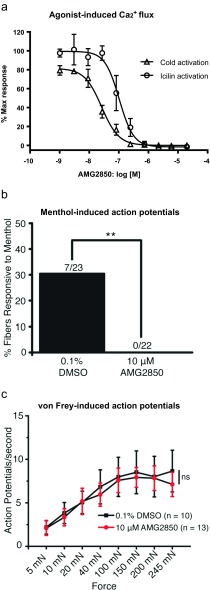
**a** AMG2850 potently inhibits both cold temperature- and icilin-induced increase in luminescence believed to reflect intracellular calcium in CHO cells stably expressing rat TRPM8. After a 2-h incubation with coelenterazine, cells were incubated with compounds for 2.5 min prior to the addition of either icilin (1 μM) or cold buffer (12 °C). Luminescence was measured by a CCD camera-based FLASH-luminometer. Concentration-response curves were generated using GraphPad Prism 4.01. Each point in the graphs is an average ± SEM of an experiment conducted in triplicate. Responses of agonists used were normalized to 100 % of maximum. **b** In a skin-nerve preparation, total percentage of C fibers responding to 300 μM menthol after incubation with vehicle or AMG2850. A response to menthol was defined as three action potentials above baseline over a 2-min interval. Incubation with AMG2850 significantly decreased the percentage of C fibers responding to menthol as compared to vehicle controls (**p < 0.005). **c** In a skin-nerve preparation, AMG2850 has no effect on C fiber firing in response to sustained mechanical force of increasing intensities. Note an increasing number of action potentials with increased force that is not altered by preincubation of AMG2850

TRPM8 antagonist hits were identified by high throughput screening of compound libraries and subsequent medicinal chemistry efforts yielded AMG2850 (Horne et al. 2014). AMG2850 blocks both icilin and cold activation of rat TRPM8 potently. The IC_50_ estimates of AMG2850 are 41 ± 8 nM against cold activation and 204 ± 28 nM against icilin activation assays (Fig. 1a). AMG2850 is >600-fold selective for TRPM8 versus TRPA1 and >100-fold selective for TRPM8 over rat TRPV1, rat TRPV3, and human TRPV4 (see Table 1).

**Table 1:** Summary of AMG2850 properties (where indicated using mean ± standard deviation)

Assay	AMG2850
rTRPM8 (cold) IC_90_ (nM)	41 ± 8
rTRPM8 (icilin) IC_90_ (nM)	204 ± 28
rTRPA1 IC_50_ (nM)	>25,000
rTRPA1 agonism IC_50_ (nM)	>25,000
rTRP(V1, V3) IC_50_ (nM)	>25,000
Rat clearance (L/h/kg)	0.47
Oral bioavailability (%)	47
B/P ratio	0.8–1.5
Protein binding	88.1 %
Rat PD ED_50_ (mg/kg)	1.8
Rat PD IC_90_ (nM)	99

### AMG2850 exhibits suitable pharmacokinetic properties for in vivo studies

AMG2850 exhibited good pharmacokinetic properties including low plasma clearance 0.47 L/h/kg and good oral bioavailability 47 % in male Sprague-Dawley rat (see Table 1). Total brain to plasma ratio of AMG2850 is in the range of 0.8–1.5 which demonstrates brain uptake and would be expected to provide coverage if there are brain TRPM8 channels that play a role in pain. We have used unbound plasma concentrations (*C*_u_; also sometimes referred to as “exposure”) in calculating the target coverage since “protein-bound” fraction of compounds are considered unavailable for TRPM8 occupancy.

### AMG2850 blocks menthol-evoked activation of C fibers in mouse

In order to better understand how the TRPM8 antagonists affect primary afferent firing, we assessed the effects of these compounds in an ex vivo skin-nerve preparation. First, it was assessed whether the compounds could block a TRPM8 agonist response. We focused on C fibers as most TRPM8-expressing afferents are C fibers (Bautista et al. 2007). C fibers treated with the vehicle control (0.1 % DMSO) responded to menthol (300 μM) with action potential firing (8.6 ± 2.9 action potentials per 2 min). In contrast, pretreatment with AMG2850 (10 μM) completely inhibited all menthol-evoked firing (*n* = 22; *p* = 0.005; Fig. 1b) demonstrating that AMG2850 completely blocks chemical activation of TRPM8 on cutaneous nerve terminals in situ.

### TRPM8 inhibition has no effect on mechanically induced firing in cutaneous C fibers

AMG2850 (10 μM) was evaluated in mechanical activation of cutaneous C fibers following a range of increasing, sustained force in the presence of the vehicle or TRPM8 antagonist. AMG2850 had no significant effect on mechanical firing in C fibers compared to vehicle controls (Fig. 1c). The C fibers treated with vehicle or AMG2850 did not differ in average conduction velocity or von Frey mechanical thresholds (data not shown). These data indicate that the TRPM8 channel does not contribute to mechanical firing following von Frey stimulation in cutaneous C fiber nociceptors.

### AMG2850 dose-dependently inhibits icilin-induced WDS in rats

**Fig. 2:**
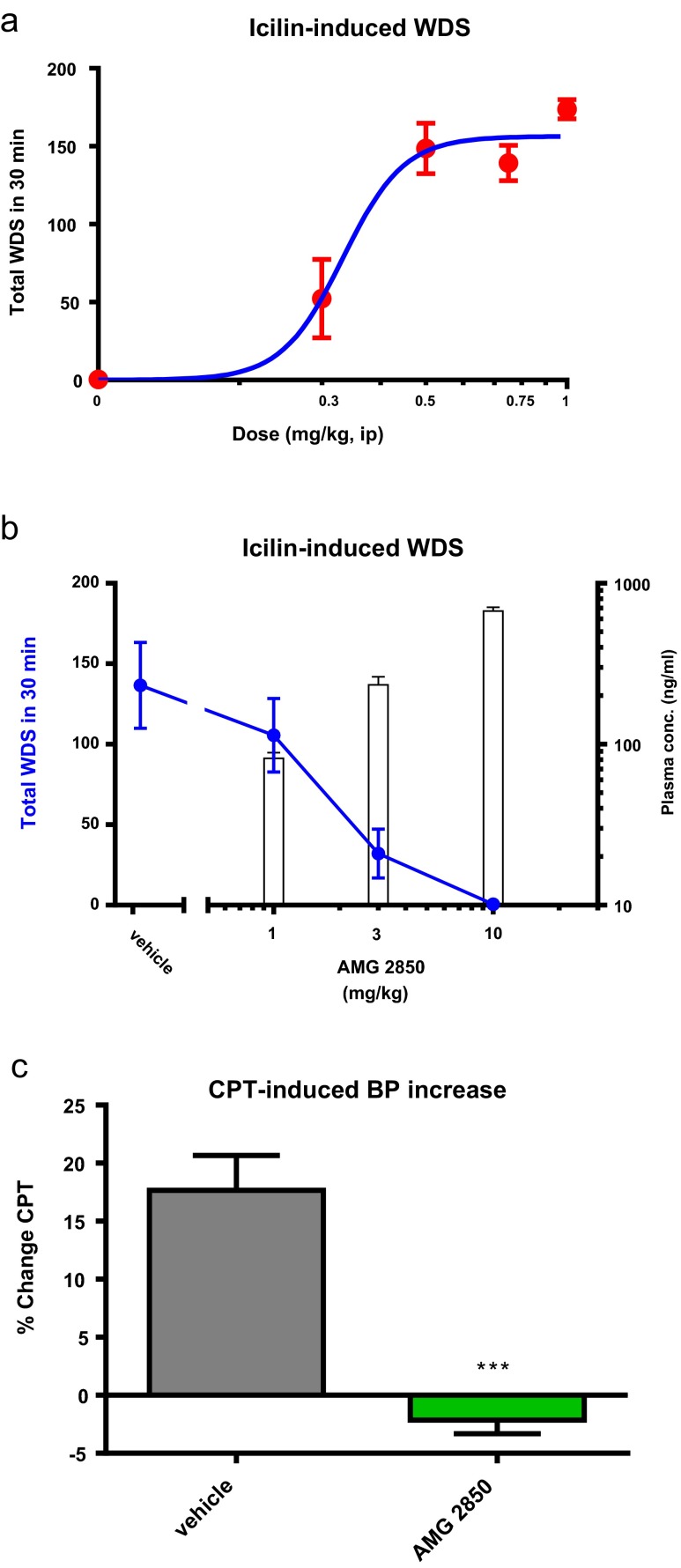
**a** Intraperitoneal injection of icilin induces wet-dog shakes (WDS) in a dose-dependent manner. **b** AMG2850 reduces WDS in a dose- and plasma concentration-dependent manner. **c** AMG2850 (10 mg/kg, p.o.) significantly and fully blocked the cold pressor effect (***p < 0.001)

To choose the icilin concentration for reversal studies, the number of WDS was evaluated following 0.3, 0.5. 0.75, and 1 mg/kg of icilin. As shown in Fig. 2a, the ED_80_ is just less than 0.5 mg/kg. In the experiments that evaluated AMG2850, 0.5 mg/kg of icilin showed a comparable 137 ± 27 WDS in the vehicle-treated groups. AMG2850 exhibited dose-dependent and full prevention of icilin-induced WDS (Fig. 2b). Full prevention occurred at 10 mg/kg with a *C*_u_ of 192 ± 26 nM and calculated unbound mean in vivo IC_90_ value of 99 nM.

### AMG2850 suppresses cold-induced increase in blood pressure

Since TRPM8 can be activated by cold in addition to icilin, we evaluated a dose near the WDS ED_90_ of AMG2850 in the cold pressor model (CPT; Fig. 2c). When tested at 10 mg/kg, AMG2850 also fully blocked the cold pressor response with a resulting *C*_u_ of 488 ± 48 nM.

### AMG2850 did not affect open field activity

**Fig. 3:**
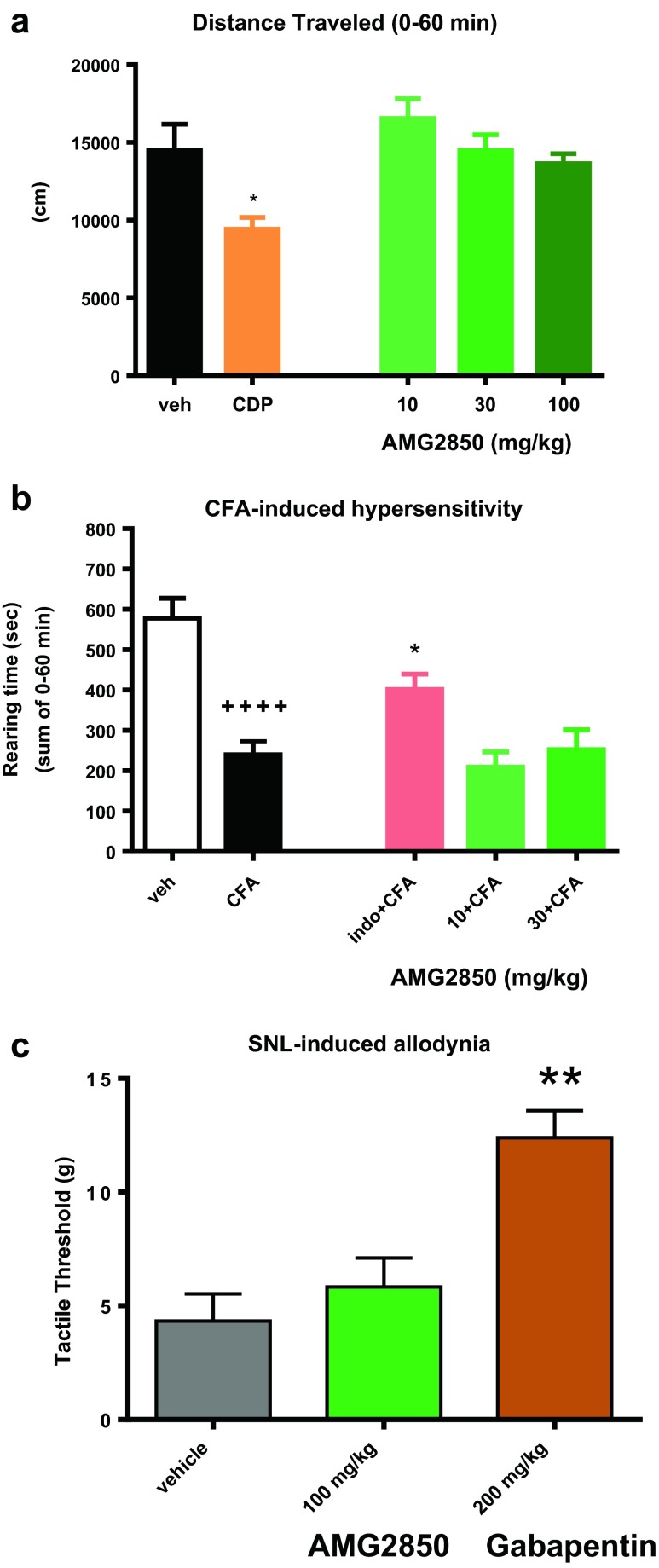
**a** AMG2850 did not affect the total distance traveled in reversed light cycle rat open field boxes (*p* > 0.05) while the positive control compound chlordiazepoxide (CDP; 5.6 mg/kg) significantly reduced distance traveled (*p < 0.05). **b** Unilateral hindpaw injection of complete Freund’s adjuvant (CFA) causes a reduction in rearing behavior. The positive control indomethacin (indo; 1 mg/kg) significantly (^++++^ *p* < 0.0001) reversed the CFA-induced reduction in rearing behavior. AMG2850 (100 mg/kg) produced no significant effect (*p > 0.05). **c** SNL produces a reduction in tactile threshold from approximately 15 g to approximately 5 g. The positive control, gabapentin (200 mg/kg), significantly reversed the reduction in tactile threshold (**p < 0.01). AMG2850 (100 mg/kg) produced no significant effect at 2.5 h post treatment (*p* > 0.05)

While assessing the potential analgesic effects of a compound, conclusions could be confounded if compounds also produce sedative or motor side effects, resulting in false positives. Thus, prior to testing in pain behavioral models, we evaluated AMG2850 in the open field assay along with a positive control of 5.6 mg/kg chlordiazepoxide (CDP; Fig. 3a). When AMG2850 was administered at 100 mg/kg (mean *C*_u_ concentration was of 2.59 μM), total distance traveled was 13,640 ± 623 cm, which was not significantly different relative to the vehicle-treated group (14,484 ± 1686 cm; *F*_3,20_ = 1.0, *p* > 0.05). As expected, total distance traveled was significantly reduced to 9423 ± 745 cm in rats receiving chlordiazepoxide (*t*_10_ = 2.8, *p* < 0.05; Fig. 3a).

### AMG2850 did not reverse CFA-induced mechanical hypersensitivity

We previously validated an automated, high-throughput assay to measure spontaneous/ongoing mechanical hypersensitivity (Youngblood et al. 2008). In this assay, rats injected with CFA to one hindpaw show a decrease in rearing behavior as measured 24 h later in an open field apparatus with a prickly floor insert. This window of presumed hypersensitivity behavior can be reversed by standard nonsteroidal anti-inflammatory drugs (Youngblood et al. 2008). In addition to automated scoring, a key advantage of this model is that reduction of pain behavior is quantified as a recovery of normal rearing instead of a decrease in an evoked response; thus, compounds that reduce general movement would not falsely be interpreted as efficacious.

In the experiment to evaluate the effects of AMG2850, there was also a significant window of hypersensitivity as measured by the rearing time response of animals administered with CFA in the hindpaw (204 ± 21 s) as compared to vehicle control in the hindpaw (525 ± 35 s; *t*_38_ = 7.9, *p* < 0.01). There was an overall significant effect on hypersensitivity (*F*_3,36_ = 4.6) with the reduction in rearing inhibited by 1 mg/kg indomethacin (388 ± 32 cm; *p* < 0.05 by Dunnett’s multiple comparisons; Fig. 3b). AMG2850 dosed at 100 mg/kg produced no significant effect on rearing time relative to vehicle (157 ± 25 s; *p* > 0.05 by Dunnett’s multiple comparisons; Fig. 3b) despite a mean *C*_u_ plasma concentration of 4.3 μM.

### AMG2850 did not reverse SNL-induced tactile allodynia

Two weeks post SNL surgery, rats exhibited a tactile threshold of 4.4 ± 1.2 g (vehicle-administered group; Fig. 3c). A logarithmic transformation of the data was performed prior to analysis, though actual *g* values are referred to in the text and shown on graphs. By Dunnett’s multiple-comparisons test relative to vehicle control, the positive control, gabapentin, significantly reversed SNL-induced mechanical allodynia with a significant increase in the threshold to 12.4 ± 1.2 g (*F*_2,31_ = 11.99, *p* < 0.05). AMG2850 at 100 mg/kg p.o. produced no significant effect on von Frey threshold relative to vehicle (*p* > 0.05) with tactile threshold of 5.8 ± 1.3 g. The mean *C*_u_ plasma concentration in this group was 2.1 μM.

## Discussion

Here, we report the pharmacology of AMG2850 as a potent and selective antagonist of TRPM8 channels in vitro and in vivo. AMG2850 demonstrated significant TRPM8 antagonism in vivo in two distinct target coverage models (icilin-induced wet-dog shake behavior (WDS) and cold-induced blood pressure increase in CPT model). AMG2850 significantly blocked menthol-induced, but not mechanically induced, activation of C fibers in situ. AMG2850 did not show efficacy in either the CFA model of mechanical hypersensitivity or the SNL-induced tactile allodynia model of neuropathic pain at plasma unbound concentrations in excess of 21-fold of an IC_90_ concentration in the TRPM8-specific WDS model. This suggests that either TRPM8 does not play a role in mechanical pain behaviors measured or a much higher target coverage is required for efficacy. Here, we discuss the results of this study in the context of antagonist exposure in vivo and TRPM8 function in different in vivo models.

### Comparison of exposure in different models: relationship to target coverage and efficacy

Protein unbound concentrations of antagonists are used for comparison between different studies. In the WDS in vivo target coverage model, resulting unbound IC_90_ value for AMG2850 is 99 nM. Compared to the WDS IC_90_ value, unbound concentration of AMG2850 in CFA and SNL models was 43- and 21-fold higher, respectively, than the WDS IC_90_ value. Lack of efficacy in both CFA and SNL models with unbound concentrations in excess of 21-fold the IC_90_ coverage in WDS model either suggests that IC_90_ coverage in WDS model is an underestimate of actual target coverage or TRPM8 does not play a role in pain behaviors measured in these models.

“Molecular sensors” such as TRPM8 (a cold temperature sensor) play a role in body temperature homeostasis by inducing autonomic and behavioral cold defenses upon activation by either cold or chemical ligands (Almeida et al. 2012; Gavva et al. 2012). WDS behavior induced by icilin probably represents a skeletal muscle-mediated heat generation mechanism and such a response may only need activation of a small subpopulation of TRPM8 channels; hence, target coverage measured in WDS model may be an underestimate relative to target coverage required in pain models in which TRPM8 channels are reportedly upregulated.

A second potential underestimation could come from the way the icilin dose is chosen for an antagonist evaluation. Typically, an ED_80_ of an agonist dose is identified based on a dose response assuming that the behavioral endpoint such as WDS is linear. However, behavioral endpoints may not be linear due to different reasons such as physical limitations, resulting in a lower ED_80_ value that may not cover the target at 80 % occupancy. This may be the case with AMG2850 where 0.5-fold of in vitro IC_90_ concentration is equivalent to WDS IC_90_ concentration.

### Role of TRPM8 antagonists on cold sensing and cold hypersensitivity

The role of TRPM8 in cold sensing and cold hypersensitivity is unequivocally proven with KO mouse studies as well as with antagonists (Knowlton et al. 2011; McCoy et al. 2011; Parks et al. 2011; Patel et al. 2014). In particular, TRPM8 antagonists representing different chemotypes such as compound 496 (Parks et al. 2011), PBMC (Knowlton et al. 2011), and M8-An (Patel et al. 2014) attenuated cold hypersensitivity after CFA or peripheral nerve injury models suggesting that TRPM8 antagonists may act as therapeutics for indications where cold hypersensitivity is an issue such as chemotherapy and other neuropathic conditions. Although we have not tested antagonists in these models, the overlap between published results and our studies is that TRPM8 antagonists cover the target in WDS model as well as suppress cold-induced increase in blood pressure (CPT model). Additionally, electrophysiological data generated using an ex vivo skin-nerve preparation demonstrates that AMG2850 is effective in blocking C fiber action potentials evoked by a TRPM8 agonist, menthol. Thus, cold-related behavioral endpoints, whether at baseline, post-inflammatory, or post-neuropathic, appear to serve as additional target coverage models for TRPM8 antagonists and are separable from efficacy models with non-cold endpoints.

### TRPM8 role in tactile allodynia

While initial reports using TRPM8 KO mice indicated that TRPM8 is not involved in mechanical sensitivity (Bautista et al. 2007; Knowlton et al. 2013), another study found increased von Frey thresholds among C fibers in exogenous skin-nerve preparations taken from TRPM8 KO mice as compared to wild-type controls (Zimmermann et al. 2011). Additionally, behavioral studies using icilin or menthol have also shown that agonist activation of TRPM8 may cause sensitization or desensitization/inhibition of responses to mechanical stimuli under naïve conditions (Brignell et al. 2008; Klein et al. 2010; Harrington et al. 2011) or after injury (Proudfoot et al. 2006; Brignell et al. 2008; Su et al. 2011; Liu et al. 2013). However, we observed no effect of AMG2850 on mechanical sensitivity using both inflammatory and neuropathic pain models, despite a robust effect on cold behaviors. Additionally, ex vivo teased fiber recordings indicated no effect of AMG2850 on mechanical sensitivity from naïve C fibers. Thus, two possibilities exist: either TRPM8 does not play a role in mechanosensation before or after injury, or AMG2850 binds TRPM8 in such a way as to inhibit ligand activation by menthol, but not its role in mechanosensation. Regardless, it can be concluded that AMG2850 is not an effective modulator of mechanical hypersensitivity or tactile allodynia, despite target coverage in vivo.

Although many have suggested TRPM8 as a therapeutic target for chronic pain, there are no examples of TRPM8 antagonists that reverse non-cold-evoked behavioral endpoints of inflammatory or neuropathic pain. One recent example is M8-An, a potent small molecule antagonist of TRPM8 shown to attenuate SNL-induced cold hypersensitivity, but was shown to be ineffective at reducing tactile allodynia (Patel et al. 2014). Similarly, AMG2850 potently blocks cold-induced physiological changes but not inflammatory- or neuropathic-induced non-cold endpoints such as mechanical hypersensitivity or tactile allodynia.

An indication for which antagonism of TRPM8 may still hold therapeutic promise is migraine, though the lack of validated preclinical migraine models leaves the promise to be revealed in the clinic. Additional indications for which new data is emerging such as urogenital diseases and pathophysiologies related to lacrimation, airway, and vasculature await further evaluation (for recent reviews, see Knowlton and McKemy 2011; Liu and Qin 2011; Malkia et al. 2011; Almaraz et al. 2014).

## Abbreviations

TRPM8: Transient receptor potential melastatin 8
TRPV1: Transient receptor potential vanilloid type 1
TRPA1: Transient receptor potential ankyrin 1
CHO: Chinese hamster ovary
CNS: Central nervous system
AMG2850: (*R*)-8-(4-(Trifluoromethyl)phenyl)-*N*-((*S*)-1,1,1-trifluoropropan-2-yl)-5,6-dihydro-1,7-naphthyridine-7(8H)-carboxamide
